# The role of serotonin in feeding and gut contractions in the honeybee^☆^

**DOI:** 10.1016/j.jinsphys.2013.12.005

**Published:** 2014-02

**Authors:** Alice S. French, Kerry L. Simcock, Daniel Rolke, Sarah E. Gartside, Wolfgang Blenau, Geraldine A. Wright

**Affiliations:** a Centre for Behaviour and Evolution, Institute of Neuroscience, Newcastle University, Newcastle upon Tyne NE1 7RU, UK; b Department of Biochemistry and Biology, University of Potsdam, Potsdam D-14476, Germany; c Department of Cell Biology and Neuroscience, Goethe University Frankfurt, Oberursel D-61440, Germany

**Keywords:** Honeybee, *Apis mellifera*, Serotonin, 5-HT, 5-HT receptor, Gut contractions

## Abstract

Serotonin (5-hydroxytryptamine, 5-HT) is involved in the regulation of feeding and digestion in many animals from worms to mammals. In insects, 5-HT functions both as a neurotransmitter and as a systemic hormone. Here we tested its role as a neurotransmitter in feeding and crop contractions and its role as a systemic hormone that affected feeding in adult foraging honeybees. We found 5-HT immunoreactive processes throughout the gut, including on the surface of the oesophagus, crop, proventriculus, and the midgut, as well as in the ventral nerve cord. mRNA transcripts for all four of the known bee 5-HT receptors (Am5-ht_1A,2α,2β,7_) were expressed in the crop and the midgut suggesting a functional role for 5-HT in these locations. Application of a cocktail of antagonists with activity against these known receptors to the entire gut *in vivo* reduced the rate of spontaneous contraction in the crop and proventriculus. Although feeding with sucrose caused a small elevation of endogenous 5-HT levels in the haemolymph, injection of exogenous 5-HT directly into the abdomen of the bee to elevate 5-HT in the haemolymph did not alter food intake. However, when 5-HT was injected into directly into the brain there was a reduction in intake of carbohydrate, amino acid, or toxin-laced food solutions. Our data demonstrate that 5-HT inhibits feeding in the brain and excites muscle contractions in the gut, but general elevation of 5-HT in the bee’s haemolymph does not affect food intake.

## Highlights

• 5-HT immunoreactive processes are present in the oesophagus, crop and midgut of *A. mellifera*.
• mRNAs for all four of the bee 5-HT receptors were expressed in the crop and midgut.
• Blockade of 5-HT receptors reduces gut contractions in the crop and proventriculus.
• 5-HT injected into the head but not the abdomen inhibits feeding.

## Graphical abstract

**Figure:**
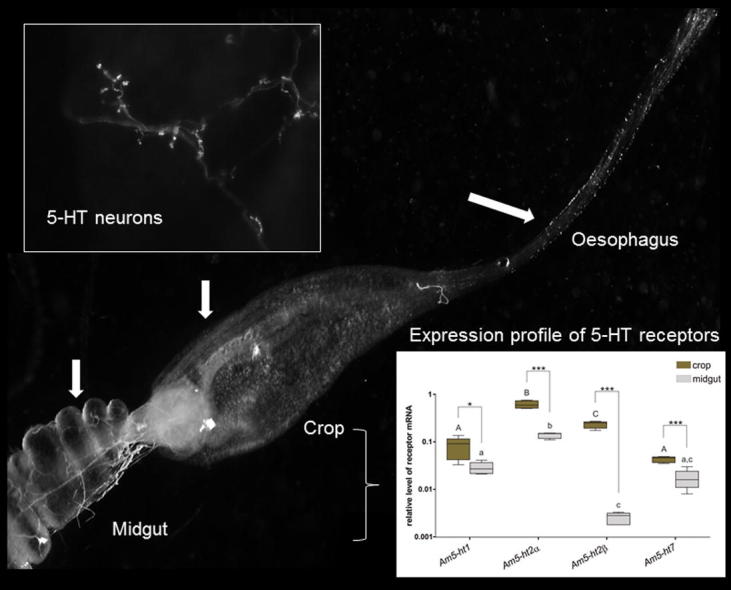

## 1 Introduction

The commencement and cessation of feeding is orchestrated by a diverse set of internal cues that provide the brain with information about nutritional state and satiety. In animals as diverse as nematodes and humans, the biogenic amine, serotonin (5-HT), is one of the key signalling molecules regulating feeding, nutrient intake and digestion (Gietzen et al., 1991; Howarth et al., 2002; Liscia et al., 2012; Marston et al., 2011; Song and Avery, 2012).

In many insects, 5-HT neurons innervate the crop and midgut (Budnik et al., 1989; Haselton et al., 2006; Molaei and Lange, 2003; Pietrantonio et al., 2001) indicating that they are likely to play an important role in the movement of food through the digestive tract. This idea has been supported by a recent study in the blowfly (*Phormia regina*) demonstrating that 5-HT applied to the crop increases muscle contractions and crop emptying rate (Liscia et al., 2012). Previous studies have also identified serotonergic varicosities in the foregut and midgut of other insect species including the kissing bug, *Rhodnius prolixus* (Lange et al., 1989), locusts, *Locusta migratoria* (Molaei and Lange, 2003) and *Schistocerca gregaria* (Johard et al., 2003), the mosquito, *Aedes aegypti* (Moffett and Moffett, 2005; Pietrantonio et al., 2001)*,* the stable fly, *Stomoxys calcitrans* (Liu et al., 2011) and the ant species, *Campanotus mus* (Falibene et al., 2012). In these species, innervation of the hindgut is often less evident. The possible presence of 5-HT neurons in the digestive tract and the functional role of 5-HT in the gut has not yet been investigated in the honeybee.

In *R. prolixus*, processes in the mesothoracic ganglion project throughout the body, and in particular, innervate the digestive tract (Lange et al., 1989; Orchard, 2006). These neurons release 5-HT directly into the haemolymph during a blood meal, but also orchestrate contractions of the crop and prime the animal’s physiology for rapid diuresis and the digestion of blood (Lange et al., 1989; Orchard, 2006). Experimental elevation of haemolymph 5-HT via direct injection into the thoracic or abdominal haemolymph in cockroaches or flies (Cohen, 2001; Dacks et al., 2003; Haselton et al., 2009) or by feeding 5-HT to ants (Falibene et al., 2012) reduces meal size, but whether this is a general mechanism for the regulation of feeding in insects remains unclear.

5-HT injected directly in the brain directly reduces the motor function of the honeybee’s mouthparts (proboscis). Studies of associative learning in honeybees indicate that 5-HT injected directly into the brain via the median ocellar tract prior to olfactory associative conditioning of the proboscis extension reflex (PER) reduces performance during conditioning (Menzel, 1999). Honeybees can also be trained to learn to withhold their proboscis to odours signalling rewards containing toxins (Wright et al., 2010). When the known 5-HT receptors in the brain are blocked using a cocktail of 5-HT receptor antagonists, bees do not learn to avoid toxins in food. They continue to extend the proboscis and feed even though the reward contains toxins, indicating that 5-HT mediates conditioned withholding of the PER (Wright et al., 2010). These two studies indicate that 5-HT is involved in the control of motor function of PER in bees, but neither has identified whether 5-HT inhibits food consumption once the proboscis is extended. In addition, no one as yet has reported whether haemolymph levels of 5-HT in the honeybee are elevated by feeding, and whether elevation of 5-HT in the haemolymph reduces food consumption by bees.

Here, we tested several hypotheses regarding the role of 5-HT in feeding the brain, gut, and ventral nerve chord of the honeybee. First, we tested whether 5-HT played a role in digestion by using immunohistochemical methods to identify 5-HT processes in the gut and ventral nerve chord. The four known 5-HT receptor homologues in bees have been measured and described from the brain (Blenau and Thamm, 2011; Schlenstedt et al., 2006; Thamm et al., 2010, 2013), but not measured elsewhere. For this reason, we also measured whether 5-HT receptors were expressed in the digestive tract and examined their role in digestion by measuring whether 5-HT affected gut contractions. Because we identified 5-HT immunoreactive processes in the ventral nerve chord, our second hypothesis tested whether systemic levels of 5-HT and/or brain 5-HT affected food intake. We first measured whether feeding elevated haemolymph 5-HT as shown in *R. prolixus* using HPLC methods. To test whether elevation of haemolymph 5-HT reduced food intake, we injected 5-HT into the abdomen and measured the consumption of sucrose solution. To verify that elevation of 5-HT in the brain but not haemolymph affected feeding, we injected 5-HT into the brain prior to assaying the total food consumption of three different types of liquid food encountered by honeybees.

## 2 Materials and methods

### 2.1 Insects

Honeybee colonies (*Apis mellifera mellifera*) were obtained from stock of the National Bee Unit (FERA, York, UK). During the months of January–March 2011 bees were maintained in an indoor flight room at a temperature of 28 °C with a 12-h light/dark cycle. During the months of May–September 2011 and 2012, bees were kept outdoors and allowed to forage freely. Adult foraging worker bees were collected in small plastic vials from outside the colony entrance. Foragers were identified as they were flying back into the colony and collected at the entrance.

#### 2.1.1 Immunohistochemistry

Using bees collected as described above, ventral nerve cords (VNC; *N* = 4), and digestive tracts (*N* = 8) were dissected in air and fixed for 1–3 h in 4% paraformaldehyde in 0.1 M phosphate buffered saline (PBS). Tissue was washed in PBS with agitation (3 changes: 10 min each) and then probed with rabbit anti-5-HT antiserum (Sigma–Aldrich, product code S5545) diluted (1:400) in 10% normal goat serum (Sigma–Aldrich, G9023) and 0.1% Triton X in PBS (NGS/PBST) for 18 h at 4 °C. Control tissues (*N* = 4 for VNC and *N* = 8 for guts) were incubated in diluent only. After incubation, probed and control tissues were first washed in PBS with agitation (3 changes: 10 min each) and incubated in biotinylated goat anti-rabbit antiserum (Vectalabs, BA-1000) in NGS/PBST (1:200) for 2 h at room temperature (RT), then washed in PBS with agitation (3 changes: 10 min each) and incubated in Fluorescein Avidin D (Vectalabs, A-2001) in NGS/PBST (1:200) for 1 h at RT in darkness. The tissue was washed a final time in PBS as before and then mounted on microscope slides under a coverslip in Vectashield mounting medium (Vectalabs, H-1500). Coverslips were sealed with clear nail polish and stored in darkness. Control tissues, which were incubated in diluent instead of primary antibody showed no positive staining, indicating that the secondary antibodies did not bind anything expressed in the tissue. Rabbit anti-5-HT antiserum (Sigma–Aldrich, product code S5545) is a commercially tested antibody previously used in insect preparations (Falibene et al., 2012), and pre-incubation of diluted antiserum with 500 μM 5-HT inhibits specific staining. Guts incubated in 1:400 concentration (*N* = 8) of primary antibody were photographed for the figures in this study, however other concentrations of primary antibody were also tested; 1:200 (*N* = 4), 1:800 (*N* = 2) and 1:1600 (*N* = 2), positive staining was observed although best images were obtained with 1:400.

#### 2.1.2 Microscopy

To obtain stacked images, specimens were examined and photographed under a Confocal Zeiss Axio Imager microscope (with apotome) using an excitatory wavelength of 488 nm. Number of *Z* slices and depth of *Z* slice interval depended on the topology and thickness of tissue. Snap shot images were obtained using a Leica DMRA fluorescent microscope with Hamamatsu GRCA-ER digital camera or Confocal Zeiss Axio Imager microscope. Images were processed using Axiovision 4.8.1 software. Light microscope images were obtained using Leica M205 C.

### 2.2 Quantitative real-time PCR

Tissue samples were collected, immediately frozen in liquid nitrogen, and stored at −80 °C until use. Total RNA was extracted using RNeasy Mini Kit (Qiagen, Hilden, Germany) and served as template for cDNA synthesis. From each sample, two independent cDNA syntheses from 250 ng total RNA were performed using SuperScript III (Invitrogen, Karlsruhe, Germany) according to the manufacturer’s instructions. Quantitative real-time PCR (qPCR) was carried out on a Rotor Gene Q (Qiagen Hilden, Germany) by using TaqMan technology with various fluorescent dyes to allow duplex measurements of receptor and reference gene expression. Fluorescent dyes used as 5′-modifications were 6-FAM-phosphoramidite (6FAM), Cy5, Cy5.5 and Yakima Yellow (YAK). BlackBerry quencher (BBQ) was attached to the 3′-end of TaqMan probes. The sequences of the primers and TaqMan probes are presented in Table 1. The PCR was performed with an initial step at 60 °C for 1 min and a denaturation step at 95 °C for 5 min, followed by 45 cycles at 95 °C for 20 s and 60 °C for 60 s. Tissue samples of individual bees were examined in triplicate. Mean copy numbers were calculated using Rotor Gene Q software (Qiagen). Receptor transcript levels were normalized to elongation factor 1α (*Amef-1α*) transcript levels (=100%) using the standard curve method. The standards covered copy numbers from 10^4^ to 10^7^.

### 2.3 Assay of crop and proventriculus contractions

**Supplementary Fig. S1:**
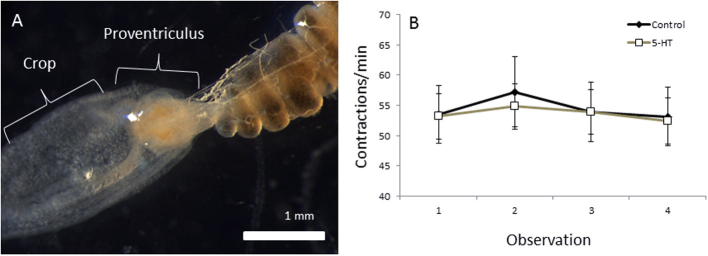
Fig. S1. Rate of contractions of the crop. (A) Light microscope photograph of the crop and 617 proventriculus, as defined for observational studies of gut contractions. (B) Application of 5-HT 618 directly to the entire gut did not alter the rate of contraction (repeated-measures ANOVA, treatment, *F*_1,33_ = 0.02, *P* = 0.890). Concentrations of 5-HT tested were 10–5, 10–7, 10–9 619 M; 620 data represent the pooled samples for all doses. *N*control = 20, Ntrt = 5/dose.

Bees were collected from the colony, immediately chill anesthetized, and then pinned to a dissecting plate dorsal side down under ‘protophormia saline’ (PPS) (Liscia et al., 2012). With the aid of a dissecting microscope, each bee was cut from the final abdominal tergite upwards towards the thorax using dissection scissors; the exoskeleton was pinned down to expose the digestive tract. After dissection, the bee was transferred to a new dissecting dish containing 5 ml PPS to cover the whole preparation; the gut remained intact within the bee. Contractions of the crop and proventriculus were observed and measured by eye under the microscope. We labelled a muscle movement in either the crop or proventriculus as a ‘contraction’ when we observed a small twitch in the wall or a complete wave of contraction along the crop wall. These contractions were labelled as arising from the crop if they occurred at the anterior end of the crop and arising in the proventriculus if they were observed posteriorly in the darkened area near the midgut (Supplementary Fig. S1).

The first observation began 1 min after the transfer; each observation lasted 1 min, with a 5 s interval between observations. Three observations were performed under PPS alone as a control. After the first 3 observations, 500 μl of solution was taken out of the body cavity and replaced with a treatment solution containing a cocktail of antagonists against the known bee 5-HT receptors (methiothepin mesylate, Sigma–Aldrich; ketanserin tartrate, Tocris Biosciences) or a control solution containing the drug vehicle (water). Water was used as the vehicle because the cocktail of antagonists was insoluble in saline. These antagonists have previously been used against 5-HT receptors in honeybees (Wright et al., 2010). The solution was applied directly above the crop and allowed to perfuse the body cavity and mix with the bath solution. The final concentration of each antagonist was 10^−4^ M or 10^−6^ M; we also tested a 10^−8^ M concentration, but it did not influence contractions. Beginning one min after the replacement of the solution, 3× one min observations were recorded with 5 s between observations. (In a pilot study, we applied 3 concentrations of 5-HT (10^−5^, 10^−7^, 10^−9^ M) to the entire gut, but did not see a measurable change in the rate of contraction of the crop or other structures (Supplementary Fig. S1B.))

### 2.4 Measurement of 5-HT in haemolymph after feeding

Adult foragers were collected and harnessed in plastic tubes, fed a 0.7 M sucrose solution to satiety and left on the bench (Wright et al., 2009). Twenty-four hours later bees were fed 5 μl of either a 1.0 M sucrose solution or a 1.0 M sucrose solution containing 0.01 M amygdalin using a Gilmont syringe. Haemolymph was extracted from the head capsules at time points 2, 5, 10, 20 and 40 min following feeding. A separate group was also measured that had not been fed (time point 0). Using a 10 μl glass capillary tube, haemolymph was acquired from a hole pierced through the exoskeleton of the head capsule near to the median ocellus. The haemolymph was immediately placed into a microcentrifuge tube containing 20 μl of 0.1 M perchloric acid on ice. Composite samples were acquired from 5 to 15 bees to a volume of ∼15 μl. The sample was brought to a final volume of 100 μl with perchloric acid, and centrifuged for 5 min at 13,000 rpm. The supernatant taken was taken off and frozen at −20 °C. Subsamples of the haemolymph were diluted to a 1:4 concentration in the HPLC mobile phase prior to analysis. Biogenic amines in 50 μl samples were analysed using HPLC with electrochemical detection (Coulochem III, ESA). A stock solution of 5-HT creatinine sulphate (Sigma–Aldrich) 10^−3^ M in 0.1 M perchloric acid was diluted to 10^−9^ M in mobile phase. 50 μl of the standard (50 fmol 5-HT) was injected every 10 samples to maintain calibration of calculated concentration. The stationary phase was a C18 reverse phase column (3 μm microsorb, 100 mm × 4.6 mm) which was maintained at 40 °C. The mobile phase (127 mM NaH_2_PO_4_, 1.5 mM octane sulfonic acid, 46.5 mM EDTA, 15% methanol, pH 3.7) was pumped through a guard cell set at +350 mV, a manual injector (Rheodyne), the column and the detector at 1.1 ml/min. Eluting 5-HT was oxidised on a porous graphite ‘frit’ flow cell with E1 set at +120 mV and E2 set at +220 mV. The resulting peak height was measured and quantified with reference to the external standard.

### 2.5 Behaviour

#### 2.5.1 Injection into head

**Supplementary Fig. S2:**
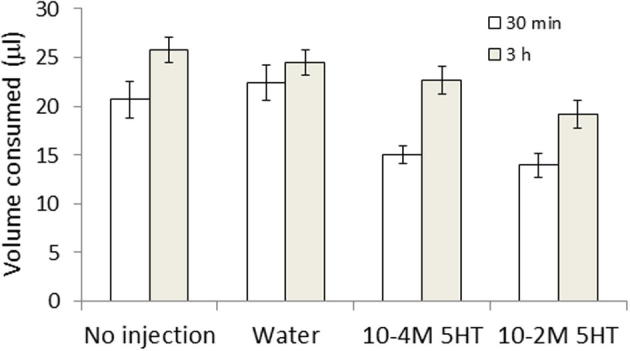
Fig. S2. In Falibene *et al*. (2012)’s study of 5-HT’s role in the consumption 621 of food by ants, 622 they found that 5-HT fed to ants was most effective at suppressing feeding 3.5 h after 623 feeding. To identify whether the time after injection affected the consumption, we tested 624 bees at 30 min and 3 h after head-injection and found that bees consumed significantly less 625 when they were tested 30 min after injection than if they had been tested 3 h later (Fig 1B, 626 2-way ANOVA, time main effect, *F*1,152 = 23.7, *P* < 0.001). At 30 min, the two doses were equally effective (lsd, *P* = 0.623); however, at 3 h, only the 10-2 627 M dose was effective at reducing feeding (lsd, 628 *P* < 0.010). *N* = 20/group.

Prior to experimentation, each bee was tested for its motivation to feed by stimulating of the antennae with 1.0 M sucrose to elicit the proboscis extension reflex (PER). Bees that did not elicit PER were excluded from the experiment. All others were split randomly into each treatment group. For the within-brain injection experiment, bees were injected into the median ocellus with 1 μl of one of the following treatments: no injection, water (injection vehicle), 10^−2^, 10^−4^ M 5-HT. Within 30 min after injection, bees were fed to satiety using a 0.2 ml Gilmont micrometer syringe with one of the following solutions: 1.0 M sucrose, 1.0 M sucrose containing a mixture of 10 essential amino acids to mimic protein (methionine, tryptophan, arginine, lysine, histidine, phenylalanine, iso-leucine, threonine, leucine, valine, each at 0.01 M for a final sum concentration of 0.1 M), or 1.0 M sucrose containing 0.1 M amygdalin. (All reagents were purchased from Sigma–Aldrich.) Satiety was indicated when the bee would no longer drink the solution and retracted its proboscis after 5 taps on the antennae with the stimulating solution. As in Falibene et al. (2012), we also tested how time after injection influenced feeding on 1.0 M sucrose solution: bees injected 30 min prior to feeding exhibited greater repression of feeding than those assayed 3 h after (Supplementary Fig. S2).

#### 2.5.2 Injection into the abdomen

For the abdominal injection experiments, bees were injected with 1 μl into the intersegmental membrane between dorsal abdominal sterna 3 and 4 (Snodgrass, 1985) (keeping the needle length parallel to the interior abdominal wall and oriented towards the petiole connecting the thorax and abdomen) with one of the following treatments: deionized water (injection vehicle), 10^−2^, 10^−4^, 10^−6^, or 10^−9^ M 5-HT. Within 30 min of injection, each bee was fed to satiety with 1.0 M sucrose and the amount consumed was recorded.

### 2.6 Statistical analysis

Analysis of variance (ANOVA) was used to analyse the food consumption experiments, 5-HT haemolymph and generalized linear modelling (GLZM) was used for the receptor expression data. 5-HT haemolymph measurements were natural log transformed prior to analysis. Gut contraction data were analysed using repeated-measures ANOVA. *Post hoc* comparisons were made using least-squares difference (lsd). All analyses were performed using the program IBM SPSS (v.19.0).

## 3 Results

### 3.1 Digestive system

**Fig. 1:**
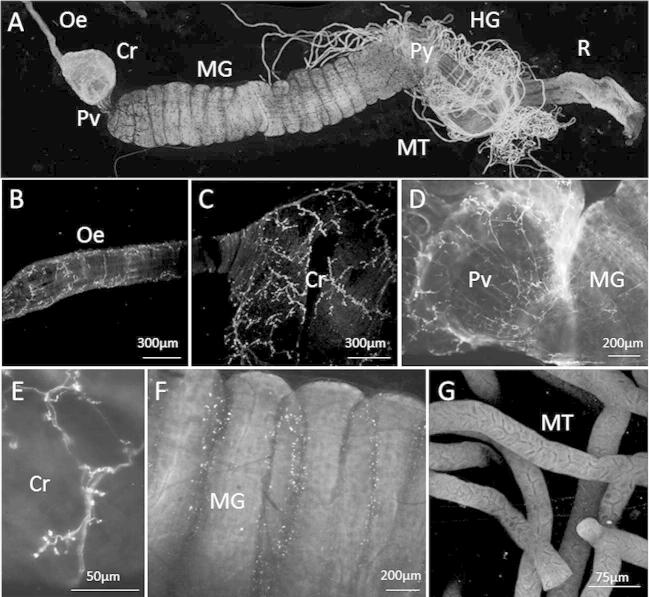
Serotonin-like innervation of the gut. (A) Shows a maximum intensity projection of the dissected honey bee gut (oesophagus (Oe) – anterior, rectum (R)–posterior) stained for 5-HT. Image is a composite of stacked and tiled images stitched together. Images acquired at 2.5× magnification. Scale bar represents 1 mm. (B) The oesophagus descends from the oral cavity where it connects to the anterior region of the crop located in the abdomen. 5-HT processes were observed on the surface of the oesophagus. Image is a composite of 20 *Z* stacks. Scale bar represents 300 μm. (C) 5-HT-like immunoreactive processes in the anterior region of the crop. Image is composite of 18 Z stacks scale bar represents 300 μm. (D) The crop and mid gut are separated by a valve called the proventriculus which is also innervated my 5-HT-like immunoreactive processes. Images is a snap shot, scale bar represents 200 μm. (E) Image shows immunoreactive processes on the crop at high magnification (50×) under oil immersion. Scale bar represents 50 μm. (F) Immunoreactive processes running circumferentially in midgut invaginations. Image is a snap shot. Scale bar represents 200 μm. (G) Malpighian tubules extend from the pylorus, a narrowing of the alimentary canal between the midgut and hindgut. No tissue specific staining was observed. Image is a composite of 14 z stacks. Scale bar represents 75 μm. Oe, oesophagus; Cr, crop; Pv, proventriculus; MG, midgut, Py, pylorus, MT, Malpighian tubules, HG, hind gut, R, rectum.

To test for 5-HT-like innervation of the gut, we examined each area of the digestive tract of the honeybee in detail after labelling with the 5-HT antibody (Fig. 1A). We identified 5-HT immunoreactive varicosities along the entire length of the oesophagus (Fig. 1B) which were continuous with the surface of the crop or honey stomach (Fig. 1C). Several 5-HT immunoreactive fibres were also identified on the crop (Fig. 1C); the proventriculus was especially densely innervated by fine processes (Fig. 1D). Many of these fibres on the crop ended in clear, bleb-like structures resembling boutons that were distributed all over the crop surface (Fig. 1E).

Dissection revealed that the midgut epithelial layer was invaginated to form a corrugated surface. Within each midgut invagination we observed a single stained process running circumferentially (Fig. 1F); these 5-HT immunoreactive processes were present in each corrugation of the entire length of the midgut. We did not find specific 5-HT immunoreactive labelling of the hypopharyngeal gland, Malpighian tubules (Fig. 1G), hindgut or the rectum.

### 3.2 Ventral Nerve Chord (VNC)

**Supplementary Fig. S3:**
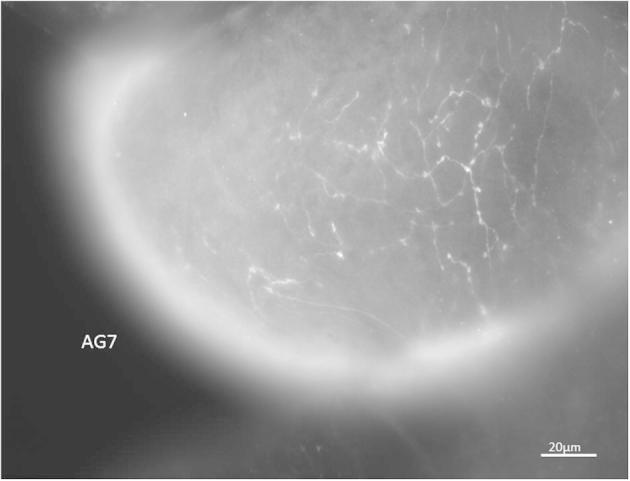
Fig. S3. Serotonergic innervation of the ventral nerve chord (VNC). The 5-HT antibody 630 labelled processes in the terminal abdominal ganglion (AG7 in diagram in Fig. 2). Scale bar 631 represents 20 μm.

**Fig. 2:**
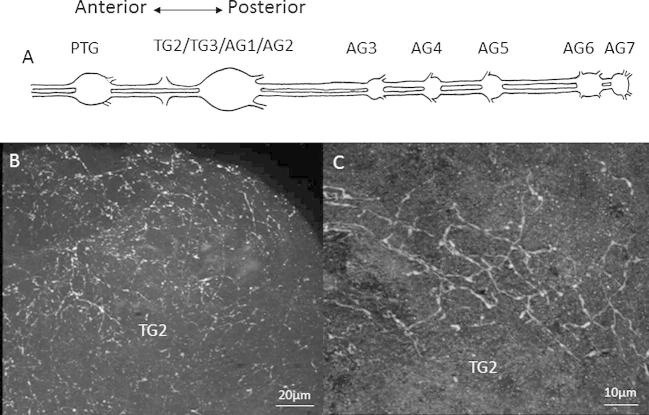
Serotonergic innervation of the ventral nerve chord (VNC). (A) A schematic showing the structure of the ventral nerve chord (VNC) and associated ganglia (TG = thoracic ganglion, AG = abdominal ganglion, not to scale); (B) 5-HT-like immunoreactive processes were distributed along the VNC, but were densest on the dorsal surface of the 2nd thoracic ganglion (TG2) (40×). Image is a composite of 30 stacked images and was taken under oil immersion. Scale bar represents 20 μm; (C) Immunoreactive processes on the dorsal surface of TG2 (63×). Image is a snapshot. Scale bar represents 10 μm.

We observed also observed 5-HT-like immunoreactive fibres throughout the VNC. In the bee, the 2nd thoracic ganglion (TG2) is fused with the 3rd thoracic ganglion and the first two abdominal ganglia (Fig. 2A, Snodgrass, 1985, Dade, 1962). We found the strongest 5-HT labelling in this structure (Fig. 2B and C). Fine networks of 5-HT-like processes were identified on the dorsal surface of the ganglion, but we did not find the same labelling of dorsal unpaired medial neuron cell bodies as observed in *R. prolixus* (Orchard, 2006). We observed similar processes in other ganglia throughout the VNC (Supplementary Fig. S3).

### 3.3 5-HT receptor expression in the crop and the midgut

**Fig. 3:**
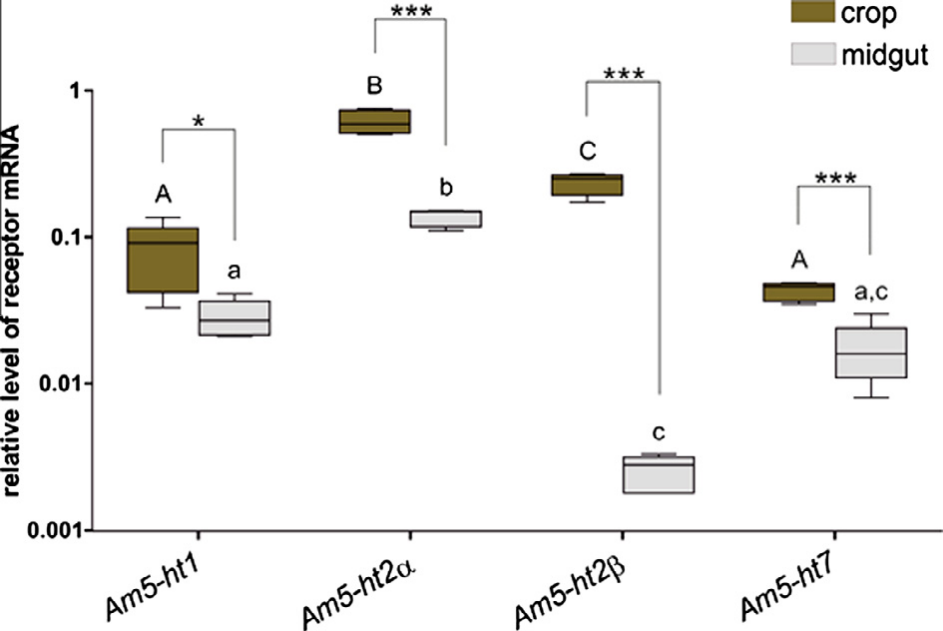
Expression patterns of 5-HT receptor genes in the crop and the midgut of adult forager honeybees determined by quantitative real-time PCR (*N* = 5/ tissue). Transcript levels were normalized to *Amef-1α* as a reference gene. Significant *post hoc* comparisons in expression of each receptor mRNA within each tissue (crop or midgut) are indicated with by letters (capital letters = crop; lower-case = midgut). Significant *post hoc* comparisons for the expression of each receptor in the crop and midgut are indicated by asterisks (indicated as ^∗^*P* < 0.05; ^∗∗∗^*P* < 0.001).

Transcripts of all 5-HT receptor genes (*Am5-ht1A*, *Am5-ht2α*, *Am5-ht2β* and *Am5-ht7*) could be detected in the crop and the midgut (Fig. 3). For all 4 receptors, the pattern of receptor mRNA expression depended on the location (2- way GLZM, receptor × tissue: $\chi_{3}^{2}=198$, *P* < 0.001). The 5-HT2 receptor transcripts exhibited greater expression levels in the crop than in the midgut. The receptor *Am5-ht2α* mRNA transcript exhibited a 15-fold greater expression in the crop than *Am5-ht2β;* in the midgut, the expression of *Am5-ht2α* was 54-fold greater in expression than *Am5-ht2β* (Fig. 3). The level of expression of *Am5-ht1A* and *Am5-ht7* was not significantly different in the crop (lsd, *P* = 0.137) or in the midgut (lsd, *P* = 0.655).

### 3.4 Activity of 5-HT in the digestive tract

**Fig. 4:**
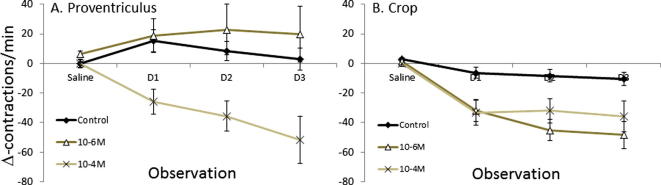
Role of 5-HT receptors in contractions of the crop and proventriculus. (A) The rate of contraction of the muscles of the proventriculus is reduced by the 10^−4^ M concentration of a cocktail of 5-HT antagonists. (B) The rate of contraction of the muscles of the crop (not including proventriculus) is reduced by the 10^−6^ M and 10^−4^ M concentrations of the 5-HT antagonists (*post hoc* lsd, 10^–6^ M: *P* = 0.001; 10–4 M: *P* = 0.008). Proventriculus: *N*_control_ = 18, *N* = 6/drug trt. Crop: *N*_control_ = 18, *N* = 6/drug trt.

Spontaneous contractions were observed in the crop and proventriculus but not the midgut. When the 5-HT receptor antagonist solution was applied, contractions in the crop and proventriculus slowed or even ceased. Prior to the application of the antagonists, the average rate of contraction of the proventriculus was 62 contractions/min (Fig. 4A), whilst the average rate of contraction of the crop was 45 contractions/min (Fig. 4B). The 10^−6^ M antagonist solution reduced contractions in the crop, but a more concentrated solution (10^−4^ M) was required to slow contractions in the proventriculus (repeated-measures ANOVA, location × treatment × time of measurement interaction, *F*_2,55_ = 6.24, *P* = 0.004). Indeed, in the proventriculus, the 10^−6^ M concentration of the antagonists tended to slightly increase contractions (Fig. 4A, repeated-measures ANOVA, treatment × time of measurement interaction, *F*_2,55_ = 7.27, *P* = 0.002). When the 10^−6^ M treatment was compared directly to the control, the effect of the drug was not significantly different (repeated-measures ANOVA, treatment, *F*_2,27_ = 0.436, *P* = 0.651; *post hoc* lsd, all comparisons *P* > 0.05).

### 3.5 Measurement of haemolymph 5-HT

**Fig. 5:**
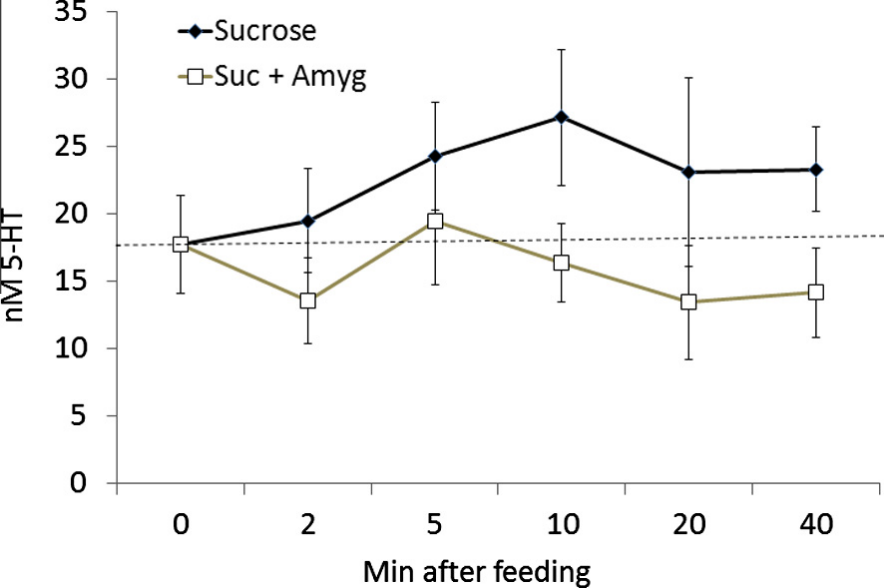
Haemolymph levels of 5-HT were higher after feeding bees 5 μl of 1.0 M sucrose (dark triangles) than when they were fed 1.0 M sucrose with 0.1 M amygdalin (open diamonds). Dashed line indicates average level of 5-HT in unfed bees. *N* = 7–11/time point for each treatment.

To test the consumption of food elevates haemolymph levels of 5-HT in the honeybee, we fed bees 5 μl of sucrose or sucrose with the nectar toxin, amygdalin, and measured 5-HT in the haemolymph collected from the head capsule at specific time points after feeding (Fig. 5). Bees fed with 1.0 M sucrose had higher concentrations of 5-HT in their haemolymph on average after feeding than bees fed with sucrose and amygdalin (2-way ANOVA, treatment main effect, *F*_1__,98_ = 7.66, *P* = 0.007). However, the concentration of 5-HT in the haemolymph of bees fed with sucrose was not significantly greater at time points after feeding (*post hoc* lsd, all *P* > 0.05). Furthermore, haemolymph 5-HT was not different at any time point after feeding with sucrose and amygdalin (*post hoc* lsd, all *P* > 0.05).

### 3.6 Injection of 5-HT into the brain suppresses feeding

**Fig. 6:**
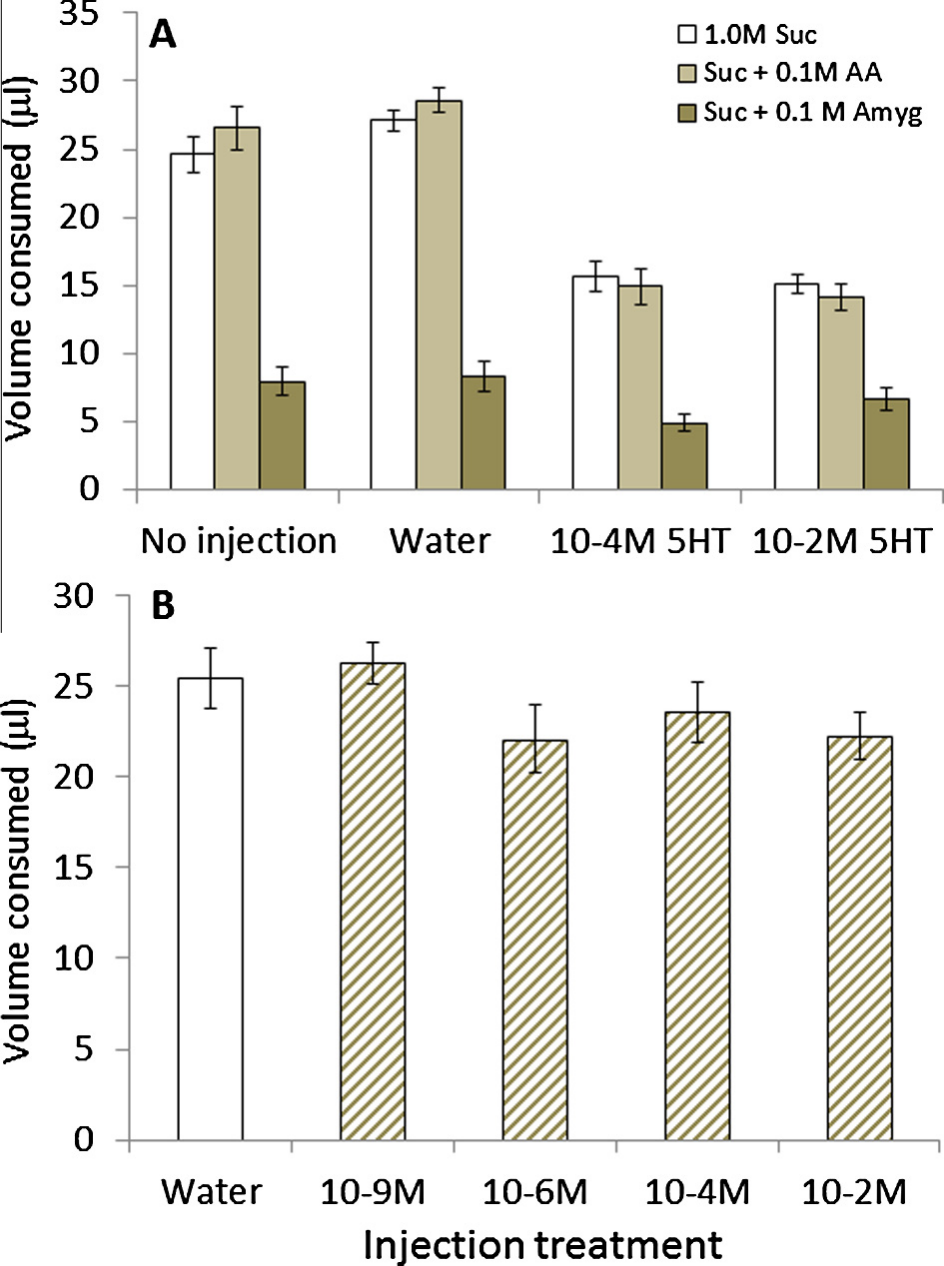
5-HT injection in the brain but not the haemolymph suppresses feeding in the honeybee. (A) Injection with 5-HT in the brain reduces meal size in bees that have been fasted for 24 h. After injection, bees were fed 1.0 M sucrose (white bars), 1.0 M sucrose with a mixture of the 10 essential amino acids (light grey bars), or a mixture of 1.0 M sucrose with 100 mM of the toxin, amygdalin (dark grey bars). *N* = 20/group. (B) Injection with 5-HT into the haemolymph of the abdomen failed to change meal size when bees were fed with 1.0 M sucrose. All concentrations on *x*-axis are of 5-HT. *N* > 25 per group.

Injection of 5-HT into the brain but not the abdomen reduced food consumption. When bees were injected into the brain via the medial ocellus with 5-HT prior to feeding and after a 24 h fasting period, the amount of food they consumed was 40–50% of what bees in the control groups (no injection or injection with vehicle) consumed (Fig. 6A). This was true regardless of the nutritional quality of the solution. The extent to which feeding was reduced by 5-HT injection, however, depended on whether the solution was carbohydrates (sucrose), a mixture of sucrose and amino acids, or a mixture of sucrose and the toxin, amygdalin (2-way ANOVA, food × treatment, *F*_6,228_ = 9.60, *P* < 0.001). The reduction in feeding was greater for bees fed with sucrose or sucrose containing amino acids than for those fed with sucrose containing amygdalin. All doses of 5-HT injected into the brain were equally effective (2-way ANOVA, 5-HT main effect, *F*_1,114_ = 3.09, *P* = 0.086). We also compared the responses of the bees in both control groups (no injection and water injection) and found no difference in these controls (2-way ANOVA, main effect, *F*_1,114_ = 0.036, *P* = 0.850).

To test whether the repression of feeding was affected by systemic levels of 5-HT, we also injected bees in the abdomen and measured feeding. We predicted that if 5-HT acted as a systemic hormone as in *Rhodnius*, elevation of 5-HT in the haemolymph after injection should repress feeding. However, unlike injection in the brain via the median ocellus, general elevation of systemic 5-HT by injection into the abdomen did not reduce the amount of sucrose solution consumed (Fig. 6B, 1-way ANOVA, *F*_4,119_ = 1.49, *P* = 0.208).

## 4 Discussion

Our data illustrate that 5-HT inhibits feeding when applied directly to the brain, but that it is excitatory in the gut. Our data show that bees have distinct serotonergic innervation of the digestive tract and ventral nerve cord as in other insects, and express all 4 known 5-HT receptors in the midgut and the crop. Additionally, we observed a difference in the haemolymph 5-HT level between bees fed with sucrose and amygdalin. However, we were unable to detect a significant elevation of 5-HT in honeybee haemolymph after feeding with sucrose. Furthermore, injection of 5-HT directly into the abdomen as a means of experimentally elevating 5-HT did not reduce feeding, but injection directly into the brain did. Below we discuss the role of 5-HT in digestion, gut motility, and the regulation of feeding circuits in the brain of the honeybee.

5-HT-like processes have been reported in the foregut of several insect species including ants (*C. mus*) (Falibene et al., 2012), fruit flies (*D. melanogaster*) (Budnik et al., 1989; Neckameyer, 2010), stable flies (*S. calcitrans*) (Liu et al., 2011), locusts (*L. migratoria*) (Molaei and Lange, 2003) and mosquitos (*A. aegypti*) (Moffett and Moffett, 2005; Pietrantonio et al., 2001). Our data adds evidence to the growing literature that indicates that 5-HT neurons and their post-synaptic receptors in these locations are involved in the control of feeding, including contractions in the insect crop (Brown, 1965; Liscia et al., 2012; Molaei and Lange, 2003). In the blowfly (*P. regina*), 5-HT causes muscular contractions of the crop and blockade of 5-HT receptors with mianserin prevents contraction (Liscia et al., 2012). However, in locusts, 5-HT relaxes the foregut, but does not cause contractions (Banner et al., 1987; Lange and Chan, 2008).

Our histological and receptor expression data also suggest that 5-HT has a role in the honeybee midgut, but direct application of excess 5-HT did not visibly affect it. However, our data are the first we know of that have reported that the proventriculus contractions are affected by blockade of 5-HT receptors. The differential sensitivity of the crop and proventriculus to the antagonists suggests that contractions in these two regions could be modulated to perform different tasks, depending on the sensitivity of the 5-HT receptors to the agonist, 5-HT. This would be important as the crop is the main organ used to store collected food such as nectar which is regurgitated by foragers on return to the colony. To regurgitate food from the crop, it would be necessary to first close the proventriculus, and then contract the crop muscles, to force fluid in the opposite direction through the digestive tract.

The pharmacology of the 5-HT receptor subtypes present in the bee has been well characterised and the binding profile of antagonist drugs has recently been determined (Blenau et al., 1995; Schlenstedt et al., 2006; Thamm et al., 2010, 2013). For example, in previous studies, methiothepin has been shown to block heterologously expressed Am5-HT_1A_ and Am5-HT_2α_ receptors (Thamm et al., 2010, 2013) and to act as an inverse agonist at Am5-HT_7_ receptors (Schlenstedt et al., 2006). Interestingly, methiothepin shows no effects at the Am5-HT_2β_ receptor (Thamm et al., 2013). Ketanserin is an antagonist of mammalian 5-HT_2A_ receptors (McKenna and Peroutka, 1989) and has been shown to block presumed 5-HT_2_ receptor agonist-mediated responses in insects (Johnson et al., 2009; Howarth et al., 2002; Gasque et al., 2013). In the bee, ketanserin seems to be a specific antagonist for the Am5-HT_2β_ receptor (Thamm et al., 2013). A previous study using a range of 5-HT agonist and antagonist drugs presented evidence that contractions in the gut of *S. frugiperda* larvae are mediated by 5-HT_2_ receptors (Howarth et al., 2002). In the present study, our cocktail of 5-HT receptor antagonists did not allow us to distinguish between the different 5-HT receptor subtypes. Based on our receptor transcript expression data and the measurement of the gut contractions, we predict that muscular contractions in the crop and gut of the bee are also mediated mainly by the Am5-HT_2_ receptors.

One of the hypotheses we tested in these experiments was that feeding alters levels of 5-HT in the haemolymph, and that this, in turn, influences the regulation of feeding as in *R. prolixus* and the flesh fly (Cook and Orchard, 1990; Dacks et al., 2003; Maddrell et al., 1991; Orchard, 2006). In these species, 5-HT is released from neurohaemal sites in the CNS (Dacks et al., 2003) and abdominal nerves associated with the mesothoracic ganglion (Lange et al., 1989; Orchard, 2006). In our immunohistochemical assays, we identified 5-HT-like processes all along the VNC, but the strongest labelling was observed a on the outer dorsal surface of the 2nd thoracic ganglion (i.e. mesothoracic ganglion). It is likely that as in other insects, the 5-HT-like neurons we identified in the VNC project to the locations we identified in the oesophagus, crop, and midgut. Unlike *R. prolixus*, however, we were unable to measure a marked elevation in haemolymph 5-HT as a result of sucrose feeding. Instead, there was a modest increase that was only observed by comparison with 5-HT measured from bees that had been fed a sucrose solution laced with the toxin, amygdalin. Our direct test of this by injecting 5-HT into the abdominal haemolymph also showed that injection did not reduce feeding on 1.0 M sucrose solution. Taken together, these data suggest that changes in haemolymph 5-HT after feeding do not act directly on circuits governing feeding behaviour in the brain of the honeybee as in *R. prolixus*.

In contrast to abdominal injection, 5-HT injected directly into brain neuropil via the median ocellus (head capsule) reduced the amount of food that bees consumed, as shown before for the bee’s PER (Menzel et al., 1999). Previous studies on the role of 5-HT in appetitive learning in honeybees support the hypothesis that 5-HT exerts inhibitory regulation of the PER and proboscis motor function. For example, when bees were injected with 5-HT prior to conditioning, they were less likely to express conditioned PER (Menzel et al., 1999) or PER towards water vapour (Blenau and Erber, 1998). Furthermore, bees that had learned to withhold the proboscis towards odours signalling food containing amgydalin failed to exhibit conditioned withholding when their 5-HT receptors were pharmacologically blocked (Wright et al., 2010). Previously, we hypothesized that 5-HT might also be a signal of malaise released by the gut or the VNC in response to stress caused by the ingestion of toxins (Wright, 2011). However, we instead found that 5-HT levels were on average lower in bees fed toxin-laced sucrose than those fed sucrose alone. Our data clarify that 5-HT does not act as a hormone released by the gut or VNC to act directly on the brain of the bee; rather, 5-HT released within the brain controls not only PER but also the amount of food consumed once the proboscis is extended.

In the brain, our data combined with previous studies suggests that 5-HT modulates food intake by inhibiting motor neurons involved in feeding. For example, in ants, 5-HT injection reduces the sucking-pumping activity of the mouthparts (Falibene et al., 2012). Immunohistochemistry studies have also revealed that serotonergic nerves innervate the mouthparts of the cockroach *Periplaneta americana* (Davis, 1987) and larval stable flies (*S. calcitrans*) (Liu et al., 2011), indicating 5-HT modulates food ingestion. It was notable that 5-HT did not completely disrupt the feeding response in the population of bees we tested, perhaps indicating that other mechanisms are necessary to completely shut down the feeding response. Instead, 5-HT reduced the total amount of food eaten by each subject.

We do not know which 5-HT receptors are involved in inhibiting feeding in bees, but in Drosophila, mutation or pharmacological blockade of 5-HT_2A_ receptor subtype inhibits feeding (Gasque et al., 2013). All of the receptors are expressed in the brain, but each is expressed in a different region. The 5-HT_1A_ receptors are expressed in the α and β lobes of the mushroom bodies, the 5-HT_7_ receptors are expressed in the mushroom body intrinsic neurons and the SOG. Both *Am5-ht2α* and *Am5-ht2β* genes are also expressed in the brain, as has been shown by qPCR experiments (Thamm et al., 2013). All of the 5-HT receptors could be involved in the regulation of feeding and the inhibition of the proboscis extension reflex (Wright et al., 2010), but we do not yet have the tools necessary to identify how they regulate these processes.

Several articles have shown that 5-HT modulates the ingestion of specific nutrients. Injection or ingestion of 5-HT reduces carbohydrate meals in ants (*C. mus*), flesh flies (*N. bullata*) and cockroaches (*R. madera*) (Cohen, 2001; Dacks et al., 2003; Falibene et al., 2012) and reduces protein meals in blowflies (*P. regina*) (Haselton et al., 2009). One study on the cockroach (*R. madera*) reported that when injected with 5-HT, cockroaches reduced their feeding on carbohydrates but not on foods containing protein (Cohen, 2001). Our study, in contrast, is the first to show that injected 5-HT suppresses feeding on a variety of food substrates in the same organism, including sucrose solutions containing a toxic substance. These data suggest that 5-HT generally inhibits the intake of food rather than affecting gustation for specific nutrients and hence the stimulation of motor output towards these nutrients.

## Figures and Tables

**Table 1:** Sequences of primers and TaqMan probes (including 5′- and 3′-modifications; see methods) used for qPCR assays and the expected length of the resulting amplicons.

Transcript	Primers and probes (5′ → 3′)	Amplicon size (bp)
Am5-ht1	Sense: ATGGTCGCCTGTCTGGTCAT	201
	Antisense: TCGTGGATTCCTCGCCTGTAT	
	Probe: Cy5-TTGAGATCGGTGACTGCCCAATATCTGT-BBQ	

Am5-ht2α	Sense: GTCTCCAGCTCGATCACGGTT	126
	Antisense: GGGTATGTAGAAGGCGATCAGAGA	
	Probe: Cy5-CGTGATCAACAACAGAGCGTTTTTCGT-BBQ	

Am5-ht2β	Sense: GAGTTTGCCACTCAGTCTGATGTACT	109
	Antisense: GCAGATTATGCTGCCGATCAAC	
	Probe: Cy5.5-TGGTGGACGGTGCCTGTCAAA-BBQ	

Am5-ht7	Sense: AATTATGTGCGACCTTTGGGTTAG	105
	Antisense: GGCTTCGTTATGGCACAGAA	
	Probe: YAK-CACAGAGATCATGCAGAGATTCAGGATGCT-BBQ	

Amef-1α	Sense: GAACATTTCTGTGAAAGAGTTGAGGC	394
	Antisense: TTTAAAGGTGACACTCTTAATGACGC	
	probe: 6FAM-ACCGAGGAGAATCCGAAGAGCATCAA-BBQ	
